# The Influence of Vicarious Fear-Learning in “Infecting” Reactive Action Inhibition

**DOI:** 10.3389/fnbeh.2022.946263

**Published:** 2022-07-22

**Authors:** Simone Battaglia, Pasquale Cardellicchio, Chiara Di Fazio, Claudio Nazzi, Alessio Fracasso, Sara Borgomaneri

**Affiliations:** ^1^Department of Psychology, Center for Studies and Research in Cognitive Neuroscience, University of Bologna, Bologna, Italy; ^2^Department of Psychology, University of Turin, Turin, Italy; ^3^IIT@UniFe Center for Translational Neurophysiology, Istituto Italiano di Tecnologia, Ferrara, Italy; ^4^Institute of Neuroscience and Psychology, University of Glasgow, Glasgow, United Kingdom; ^5^Istituto di Ricovero e Cura a Carattere Scientifico (IRCCS) Fondazione Santa Lucia, Rome, Italy

**Keywords:** SARS-CoV-2, vicarious fear-learning, action inhibition, negative emotion, stop-signal task (SST)

## Abstract

Since the dawn of cognitive neuroscience, emotions have been recognized to impact on several executive processes, such as action inhibition. However, the complex interplay between emotional stimuli and action control is not yet fully understood. One way to measure inhibitory control is the stop-signal task (SST), which estimates the ability to cancel outright an action to the presentation of a stop signal by means of the stop-signal reaction times (SSRTs). Impaired as well as facilitated action control has been found when faced with intrinsic emotional stimuli as stop signals in SSTs. Here, we aimed at investigating more deeply the power of negative stimuli to influence our action control, testing the hypothesis that a previously neutral stimulus [i.e., the image of the severe acute respiratory syndrome coronavirus 2 (SARS-CoV-2)], which has been conditioned through vicarious fear learning, has the same impact on reactive action inhibition performance as an intrinsically negative stimulus (i.e., a fearful face or body). Action control capabilities were tested in 90 participants by means of a SST, in which the stop signals were represented by different negative stimuli. Results showed that the SARS-CoV-2 image enhanced the ability to suppress an ongoing action similarly to observing fearful facial expressions or fearful body postures. Interestingly, we found that this effect was predicted by impulsivity traits: for example, the less self-control the participants had, the less they showed emotional facilitation for inhibitory performance. These results demonstrated that vicarious fear learning has a critical impact on cognitive abilities, making a neutral image as threatening as phylogenetically innate negative stimuli and able to impact on our behavioral control.

## Introduction

Emotional information is integral to everyday life and impacts a variety of cognitive abilities including response inhibition, a critical skill to enhance fitness to the social environment. The proper neural networking between behavioral domains such as emotion and cognition and subsequently developed coping behaviors are essential mechanisms to mental well-being. The malfunction due to unwanted experience, anticipation, fear, pain, chronic stress, neuroinflammation, and maldevelopment may lead to development of mental illnesses (Spekker et al., 2021; Tanaka et al., 2021, 2022; Martos et al., 2022). The effects of emotion on response inhibition, however, are inconsistent, with studies collectively showing that emotion can impair, facilitate, or have no effect on action control (Battaglia et al., 2021). Recent theories (Aron, 2011; Mirabella, 2021) propose that inhibitory control is not a single executive function, but it encompasses two domains: reactive and proactive inhibition. Proactive inhibition is the ability to adapt the motor strategy flexibly according to *a priori* knowledge, while reactive inhibition refers to the outright stopping in response to an unexpected change in the context. The ability to inhibit prepotent responses that has already been initiated can be investigated using stop-signal tasks (SSTs), designed to provide a sensitive measure of the time taken by the brain to inhibit or suppress inappropriate motor responses (Logan and Cowan, 1984; Verbruggen et al., 2019). The SST requires participants to respond to a Go stimulus and to interrupt the ongoing response when a Stop signal is presented. To measure the participant’s performance on the SST, the stop-signal reaction time (SSRT), an index of reactive inhibition, is computed based on Logan and Cowan’s notion (Logan and Cowan, 1984). Estimated SSRT gives the measure of the duration of the inhibitory process, with a lower value indicating a more rapid ability to respond to a Stop signal (Cai et al., 2015). SST studies have reported that, in some cases, emotional stimuli impaired response inhibition compared to neutral images (Verbruggen and De Houwer, 2007; Herbert and Sütterlin, 2011; Kalanthroff et al., 2013; Rebetez et al., 2015; Mancini et al., 2022), while in other studies emotional stimuli facilitated response inhibition compared to neutral cues (Pessoa et al., 2012; Senderecka, 2016; Choi and Cho, 2020). On the other hand, some studies reported no differences between response inhibition for emotional versus neutral stimuli (Sagaspe et al., 2011; Patterson et al., 2016) but consider evidence from Go/No-Go task 15. Given the daily relevance of emotion processing and response inhibition, a deeper understanding of how these constructs interact is highly desirable.

In an attempt to solve the issue of contrasting results, the dual competition framework has been proposed, which assumed that the emotional content influences both perceptual and executive control processes (Pessoa, 2009). According to this framework, the effect of emotion on cognition depends on the intensity level of the affective information. Task-relevant stimuli of mild intensity improve executive control since they increase goal-directed behavior, whereas high-intensity stimuli attract resources available for the task and hence disrupt executive processes. To test this hypothesis, Pessoa et al. (2012) evaluated the impact of emotional low-intensity stimuli (faces) on response-inhibition performance. The results showed that SSRT was affected by the emotion, so that shorter SSRT were recorded in emotional conditions with respect to neutral ones, suggesting that participants were better at inhibiting the responses with emotional stimuli, which could be fearful or happy faces. In a second experiment, a stronger emotional stimulus (a fear-conditioned auditory stimulus) was used as Stop-signal. In line with the idea that the intensity of the Stop signal may influence the effect of emotional stimuli, SSRT was longer during the fear-conditioned condition compared to the neutral condition, demonstrating that it was harder to inhibit the behavioral response during the former highly arousing condition. The authors interpreted these opposite effects by suggesting that emotion can either enhance or impair cognitive performance, likely as a function of the emotional saliency of the stimuli involved. Stop signals of different intensities may, for example, impact separable mechanisms contributing to the observed behavior. However, the two Stop signals were fundamentally different in perceptual characteristics (i.e., a facial stimulus with a fearful expression versus an auditory tone). Moreover, negative faces are phylogenetically negative innate stimuli, while the threateningness of the auditory tone was acquired through a fear conditioning procedure. Therefore, it is unclear whether it was the intensity of the stimuli, rather than the fact of being intrinsically negative or not, that differently affected the stopping performance.

To overcome these possible confounding elements/factors/scenarios, here we aimed at investigating the impact of a stylized image of the severe acute respiratory syndrome coronavirus 2 (SARS-CoV-2) on the ability to inhibit our actions. Such image was conditioned by means of vicarious fear-learning (Olsson and Phelps, 2007; Debiec and Olsson, 2017) through massive media exposure, but it has similar perceptual characteristics to a face, as well as the same intensity (see section “Stimuli Validation”). As intrinsic negative images, we used a fearful facial expression, as in Pessoa et al. (2012), and fearful body postures as an additional negative stimulus, since no previous studies employed emotional body stimuli as stop signals. However, due to fact that both stimuli are intrinsically negative, no differences are expected in their ability to impact the action control. The SARS-CoV-2 infection causes coronavirus disease 2019 (COVID-19), which is a new, rapidly spreading, and highly contagious pandemic infectious disease (Sohrabi et al., 2020). As of 1st October 2020, COVID-19 had affected over 235 countries with a total of 37.7 million confirmed cases, and 1 million deaths worldwide (World Health Organization, Geneva, Switzerland). Critically, Italy was the first Western country to experience a large number of COVID-19 cases in early 2020, and therefore adopted strict lockdown measures. Thus, the SARS-CoV-2 pandemic is not only a threat to physical health but is also having severe impacts on mental health (D’Agostino et al., 2020). Pandemics induce high levels of stress and result in mental health problems associated with a variety of psychiatric and psychological conditions such as depression, anxiety, and post-traumatic stress disorder symptomatology (Reissman et al., 2006; Lee et al., 2007; Pfefferbaum et al., 2012; Lei et al., 2020; Shi et al., 2020; Vindegaard and Benros, 2020; Lisi et al., 2021; Veer et al., 2021). Thus, the urgent involvement of mental health science in the SARS-CoV-2 pandemic is crucial, and it has also been pointed out that such investigation would be necessary to better prepare populations and health systems for future pandemics or potential further lockdowns (Holmes et al., 2020; Kisely et al., 2020; Lai et al., 2020; Sohrabi et al., 2020; Zhang and Ma, 2020). To our knowledge, the COVID-19 pandemic has not only resulted in physical conditions, but also social, psychological, and economic consequences have been observed globally: fear of COVID-19 infection has been reported as the main psychological stressor during the disease outbreak, with various domains of worries related to this fear, like fear of oneself or their family members getting infected, fear of having economic losses and being unemployed, avoidance behaviors toward gaining knowledge about the pandemic or fear of making decisions on showing or not showing actions (like whether to visit parents or not) (Taylor et al., 2020; Balbuena and Monaro, 2021; Quadros et al., 2021).

Herein, we investigated, for the first time, the impact of a social conditioned stimulus (i.e., the image of the SARS-CoV-2) on the ability to influence our behavior. In order to evaluate whether vicarious fear learning is able to make the intrinsically neutral image of SARS-CoV-2 as threatening as a phylogenetically innate stimulus, we compared the influence of the SARS-CoV-2 image with phylogenetically innate stimuli (i.e., fearful faces and body postures) in terms of the ability to influence response inhibition. Additionally, since it has been found that personality traits like trait anxiety (Avila and Parcet, 2001; Neo et al., 2011; Hsieh et al., 2022) and impulsivity (Logan et al., 1997; Avila and Parcet, 2001; Bari and Robbins, 2013; Pawliczek et al., 2013) may impact the ability to suppress an ongoing action, we also tested whether such personality traits have an influence on the action control when faced with negative stimuli. The present study constitutes one of the first evidences in neuroscience and psychological science of how the COVID-19 pandemic has affected human behavior.

## Materials and Methods

### Participants

A total of 140 right-handed healthy volunteer adults participated in the present study, 50 of whom took part in the pilot study to validate the visual stimuli, while the remaining 90 were involved in the main experiment (i.e., the SST). All participants were recruited using a snowball sampling approach, started on 1st April 2020, *via* social media, mailing lists, and a general media campaign, and ended on 31st August 2020. This period corresponds to a phase in which the spread of SARS-CoV-2 was uncontrollable and strict lockdown measures were in place in Italy as well as in almost all European countries, with a death rate over of 10% (the second most affected country after China) over the same period; overall 275,000 cases and 35,000 deaths were recorded in Italy (World Health Organization, Geneva, Switzerland). Prior to participation, subjects declared that they had no history of neurological, psychiatric, or SARS-CoV-2 diagnosis, and none of the participants was regularly taking any medication affecting the central nervous system. All participants had normal or corrected-to-normal vision. In the main SST experiment, due to the high numbers of trials, participants were randomly divided into 3 groups: 30 were assigned to the SARS-CoV-2 group, 30 to the Fear-Face group, and 30 to the Fear-Body group. The number of participants was determined based on a power analysis, which indicated that a sample size of 30 participants is necessary to achieve a statistical power (1 − β) of 0.99 [two-tailed α = 0.01; effect size *f* = 0.4 (Pessoa et al., 2012); number of measurements = 2; correlation = 0.5, analysis performed with G*Power software (Faul et al., 2007)]. Finally, the groups were matched for age [*F*(2,87) = 1.864; *p* = 0.16; η*_*p*_*^2^ = 0.041], years of education [*F*(2,87) = 2.629; *p* = 0.08; η*_*p*_*^2^ = 0.057], and gender [c^2^(2, *N* = 90) = 1.216; *p* = 0.54; see Table 1 for further demographic data].

**TABLE 1:** Demographic data.

Group	Age	Education	Gender
SARS-CoV-2	22.33 ± 2.02	14.20 ± 1.81	*F* = 19; *M* = 11
Fear-Face	22.63 ± 3.63	14.30 ± 1.82	*F* = 16; *M* = 14
Fear-Body	23.63 ± 2.25	15.23 ± 2.13	*F* = 20; *M* = 10

*Age and education are reported as mean ± SD, expressed in years. Gender is reported as the number of female and male participants.*

Furthermore, different personality states of the participants were investigated, as previous studies have shown that SST performance, as well as reactive action inhibition, may be influenced by psychological or psychiatric conditions (i.e., anxiety, depression, impulsivity) (Pessoa et al., 2012; Legrand and Price, 2020; Battaglia et al., 2021). Subjective levels of anxiety were measured through the State-Trait Anxiety Inventory (STAI; Trait-Scale-Y2) (Spielberger et al., 1983) and subjective levels of impulsivity were measured by the Barratt Impulsiveness Scale-11 (BIS-11) (Patton et al., 1995). The STAI-Y2 consists of a 20-item self-report questionnaire providing an assessment of anxiety and evaluates how often respondents experience anxiety. The BIS-11 is a questionnaire designed to assess the personality construct of impulsiveness, it is composed of 30 items assessing common impulsive or non-impulsive behaviors. Finally, we administered the Hospital Anxiety and Depression Scale (HADS) (Zigmond and Snaith, 1983) to exclude participants with high levels of anxiety and depression in our sample. The HADS is a 14-item questionnaire designed to assess levels of anxiety and depression that a person is experiencing, it consists of 7 questions for anxiety and 7 for depression. The three groups did not show any significant difference in terms of anxiety [STAI-Y2: *F*(2,87) = 0.508, *p* = 0.60, η*_*p*_*^2^ = 0.012; HADS-anxiety: *F*(2,87) = 0.852, *p* = 0.43, η*_*p*_*^2^ = 0.019], HADS-depression [*F*(2,87) = 1.164, *p* = 0.32, η*_*p*_*^2^ = 0.032], and BIS-impulsivity [*F*(2,87) = 0.698, *p* = 0.50, η*_*p*_*^2^ = 0.016] scores (see Table 4 for further details). Furthermore, we ensured that all tested participants had never been diagnosed with COVID-19, thus avoiding any personal bias or physical condition. Data collection was anonymous, and all participants gave their informed consent electronically through our online platform before the task. Data were hosted and stored on a private server and were password protected and accessible only by the corresponding authors. The study was conducted in accordance with the ethical principles of the World Medical Association Declaration of Helsinki and was approved by the Ethics Committee of the Department of Psychology of the University of Bologna.

### Stimuli Validation

A pilot study was conducted to ensure that the image of SARS-CoV-2, of a fearful face expression, and of a fearful body expression, were considered equally negative and more negative than other neutral stimuli counterparts, respectively, a fractal, a Neutral-Face, and a Neutral-Body posture. To this aim, 50 healthy participants (22 female; mean age ± SD: 28.1 ± 4.2 years) were recruited for the stimuli validation and were not involved in the subsequent SST (i.e., main experiment). Participants were initially shown all images and had to make explicit recognition of the images based on multiple proposed alternatives. The outcome was that the images were correctly associated with the appropriate alternative (see Table 2 for further details). The participants were then presented with different questions to rate the stimuli’s perceived fear and arousal. The order of the different judgments was randomized across participants. Participants used an electronic 10-point Likert scale ranging from 1 (none) to 10 (extremely). To investigate differences in perceived fear between stimuli a 2 × 3 analyses of variance (ANOVA) with Emotion (Negative/Neutral) and Stimuli (SARS-CoV-2/Fear-Face/Fear-Body) as within-subject factors was carried out. The analysis revealed the main effect only of Emotion [*F*(1,2) = 218.44, *p* < 0.001, η*_*p*_*^2^ = 0.59] and Bonferroni *post hoc* comparison showed significantly higher rates for negative (5.77 ± 2.91) than neutral stimuli (2.1 ± 1.67; *p* < 0.001; see Table 2 for further details). No other main effects or interactions were found to be significant (all *F* < 0.86; *p* > 0.42; η*_*p*_*^2^ < 0.01), indicating that the three stimuli were comparable for the amount of fear they conveyed. Similarly, to investigate differences in arousal between the three stimuli a 2 × 3 ANOVA with Emotion (Negative/Neutral) and Stimuli (SARS-CoV-2/Fear-Face/Fear-Body) as within-subject factors was carried out. The analysis again revealed the main effect only of Emotion [*F*(1,2) = 176.73, *p* < 0.001, η*_*p*_*^2^ = 0.54] and Bonferroni *post hoc* comparison showed significantly higher rates for negative (6.42 ± 2.56) than neutral stimuli (3.53 ± 2.03; *p* < 0.001; see Table 2 for further details). No other main effects or interactions were found to be significant (all *F* < 2.08; *p* > 0.12; η*_*p*_*^2^ < 0.02).

**TABLE 2:** Stimuli validation.

Stimuli	Accuracy (%)	Perceived fear	Arousal
SARS-CoV-2	98	6.08 ± 3.17	6.92 ± 2.76
Fractal	92	2.24 ± 1.82	3.92 ± 2.06
Fear-Face	98	5.68 ± 2.79	6.38 ± 2.53
Neutral-Face	98	2.26 ± 1.71	3.24 ± 2.01
Fear-Body	96	5.56 ± 2.38	5.98 ± 2.33
Neutral-Body	90	1.81 ± 1.42	3.44 ± 2.01

*Ratings are reported as mean ± SD for each stimulus presented. Participants used an electronic 10-point Likert scale ranging from 1 (none) to 10 (extremely).*

These results showed that the three negative stimuli used were perceived as similarly negative and produced compatible affective reactions, independently from the actual content of the stimulus. Moreover, each one of these negative images was perceived as more arousing and more threatening than its neutral control. Thus, the negative stimuli and their neutral counterparts were controlled and balanced.

### Stimuli

**FIGURE 1:**
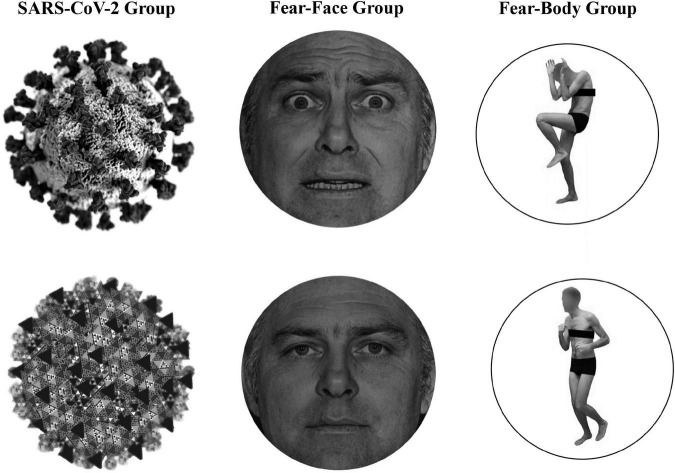
Stimuli used as stop-signal stimuli. In the SARS-CoV-2 group stimuli consisted of a stylized black and white image of the virus COVID-19 and an image of a black and white fractal with irregular outlines, acting as control neutral stimulus. In the Fear-Face group stimuli consisted of two different face pictures, showing a fear and neutral expression, which were selected from the Ekman set (Ekman and Friesen, 1976). In the Fear-Body group, stimuli consisted of two different body pictures with fearful and neutral expression, previously used in Borgomaneri et al. (2015a,b,c, 2017).

In the main experiment, Go-stimuli consisted in the presentation of a black arrow pointing left or right, while the Stop stimuli presented to the SARS-CoV-2 group were a stylized black and white image of the virus COVID-19 and an image of a black and white fractal with irregular outlines that recalled the shape of the virus and acted as a control neutral stimulus (see Figure 1). Two different face pictures (i.e., fearful and neutral expression) selected from the Ekman’s Pictures of Facial Affect (POFA) set (Ekman and Friesen, 1976) were used as Stop-signals and were presented to the Fear-Face group (see Figure 1). Two different body pictures (i.e., fearful and neutral expression) previously validated in Borgomaneri et al. (2015a,b,c, 2017) were used as Stop-signals for the Fear-Body group (see Figure 1). Stimuli were edited to have the same shape, surface, complexity, colors, and contrast ratio with Blender (Blender Foundation, Amsterdam, Netherlands) and Adobe Photoshop CS6 software (Adobe, San Jose, CA, United States).

### Online Task Features

A classical SST was employed to measure response inhibition (Vince, 1948; Lappin and Eriksen, 1966; Logan, 1994). We created an online and accessible web-version of the SST running on the Internet on both laptop/desktop computers. Thus, the task was developed in-house using the jsPsych library version 6.1.0 (De Leeuw, 2015), which is based on JavaScript ES6^1^ of a classical custom-made SST running local-only. Recent studies suggested that response time measurements using jsPsych are comparable to those taken with standard lab-software (Reimers and Stewart, 2015; De Leeuw and Motz, 2016; Hilbig, 2016; Pinet et al., 2017). Therefore, our SST online web-version relies on the jsPsych library with custom modifications to improve the interface and user experience. The script’s code was compiled with JetBrains IntelliJ IDEA 2020.1.1 software.^2^ The task is hosted on a Google Firebase hosting,^3^ and its code is deposited on a GitHub repository.^4^ Finally, the data collected during the experiment were automatically saved on an external server and the storing was made using a PHP script,^5^ which was password-protected and accessible only by the authors.

### Stop-Signal Task

**FIGURE 2:**
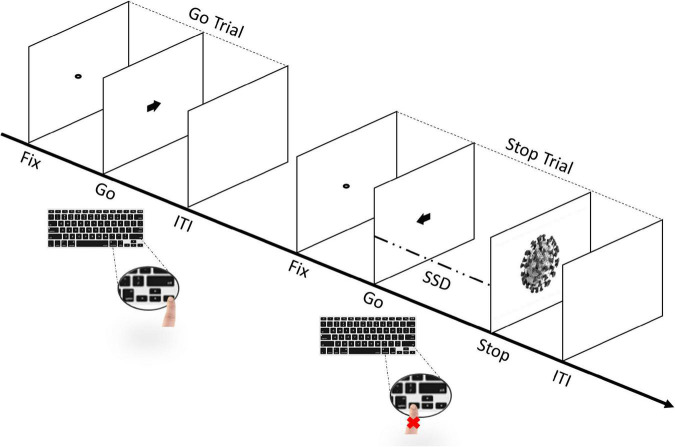
Sequence of trials in the stop-signal task (SST). The experimental task includes both Go- and Stop-trials (Lappin and Eriksen, 1966; Logan and Cowan, 1984; Logan et al., 2014; Verbruggen et al., 2019). Participants perform a short practice block and, immediately afterward, four experimental blocks. Each block includes a total of 128 trials, of which 96 are Go-trials (75%) and 32 are Stop-trials (25%). In Go-trials, participants respond to the Go-task (i.e., the direction of the arrow that appears on the screen) by pressing the corresponding arrow key on the keyboard. In Stop-trials, the arrow is followed by a “Stop” signal after a variable stop-signal delay (FIX, fixation duration; SSD, stop-signal delay; ITI, intertrial interval), instructing participants to suppress the imminent Go response. The initial value of the SSD was set to 150 ms and adjusted individually and dynamically throughout the experiment (i.e., staircase procedure), so that, if participants successfully inhibited their response on a Stop-trial, the SSD was increased by 50 ms in a subsequent Stop-trial, while if they failed to withhold their motor response, the SSD was reduced by 50 ms in a subsequent Stop-trial.

The experimental task consisted of a simple reaction time (RT) task, which included both Go- and Stop-trials (Lappin and Eriksen, 1966; Logan and Cowan, 1984; Logan et al., 2014; Verbruggen et al., 2019). Participants started by performing a short practice block (approximately 3 min, 32 trials) to familiarize themselves with the task. Immediately afterward, they performed four experimental blocks that constituted the main task. Each block was composed of a total of 128 trials, of which there were 96 Go-trials (75%) and 32 Stop-trials (25%). Thus, during the whole task, each participant was presented a total of 384 Go-trials and 128 Stop-trials. In each block, the Go- and Stop-trials contained stimuli in equal proportion. Each trial started with the presentation of a black dot centered on a blank white screen for 800 ms (i.e., fixation point) and ended with an empty blank white screen for 1600 ms, acting as an inter-trial interval (ITI). In the Go-trials, participants had to perform with their right hand, a Go-task by pressing the left key when a black arrow pointing to the left appeared, or the right key when the arrow pointing to the right appeared, for 100 ms. On the other hand, the Stop-trials were identical to the Go-trials, except that a picture of a stimulus (i.e., Stop-signal) was presented for 70 ms, after a variable stop-signal delay (SSD) relative to the onset of the Go-stimulus (i.e., the arrow), instructing participants to suppress the imminent Go response (see Figure 2). The initial value of the SSD was set to 150 ms and adjusted individually and dynamically throughout the experiment (from a minimum of 50 ms to a maximum of 650 ms), a procedure referred to as “staircase.” So that, if participants successfully inhibited their response on a Stop-trial, the SSD was increased by 50 ms on a subsequent Stop-trial, while if they failed to withhold their motor response, the SSD was reduced by 50 ms on a subsequent Stop-trial (Logan et al., 1997; Verbruggen and De Houwer, 2007; Pessoa et al., 2012; Senderecka, 2016; Verbruggen et al., 2019; Legrand and Price, 2020; Stockdale et al., 2020). Importantly, the staircase was independent within-subject, as the SSD was adjusted separately for each stimulus (i.e., the staircase for one stimulus was calculated independently from the next stimulus in each participant) to ensure successful inhibition in approximately 50% of the Stop-trials for each stimulus (Band et al., 2003; Verbruggen et al., 2019; Hilt and Cardellicchio, 2020). Participants were instructed to respond as quickly and accurately as possible to the arrow and were asked to inhibit their response upon viewing a stimulus which followed the initial Go-stimulus that appeared on the screen. However, they were also instructed that sometimes it might not be possible to successfully inhibit their response and, in such cases, they should continue to perform the task irrespective of having made an error (Pessoa et al., 2012; Verbruggen et al., 2019). Furthermore, participants were asked not to hesitate or slow down to avoid increasing the chances of stopping. Overall, our task was designed based on the recommendations of Verbruggen et al. (2019). Finally, participants were automatically redirected to the personality traits questionnaires.^6^

### Data Processing and Analysis

To measure the participants’ performance on the SST, SSRT, an index of reactive inhibition, was estimated based on Logan and Cowan’s notion of the race-model (Logan and Cowan, 1984). SSRT is the overall latency of a chain of processes involved in stopping a response, including the detection of the Stop-signal. However, prior to analyzing SSRT, the reliability of the overall performance of the participants in the task was verified by calculating the inhibition rate, which must be around 50% (Band et al., 2003; Logan et al., 2014; Matzke et al., 2018; Verbruggen et al., 2019). Subsequently, data collected in this experiment were processed to estimate SSRT according to “the consensus guide to capturing the ability to inhibit actions and impulsive behaviors in the stop-signal task” (Verbruggen et al., 2019). Accordingly, data were analyzed adopting the integration method with the replacement of Go-omissions. In particular, the point at which the Stop process ended is estimated by “integrating” the RT distribution and finding the point at which the integral is equal to “p(respond| signal).” The ending time of the stop process corresponded to the *n*th RT, where *n* = the number of RTs in the RT distribution of Go trials multiplied by “p(respond| signal).” Also, to determine the *n*th RT, all Go trials with a response were considered, including Go-trials with a choice error and Go-trials with a premature response. It is important to highlight that omissions (i.e., Go-trials in which participants did not respond before the end of the trial) were assigned the maximum RT to compensate for the lack of response. Moreover, premature responses in unsuccessful Stop-trials (i.e., responses executed before the Stop-signal is presented) were included in calculating “p(respond| signal)” and mean SSD. This version of the integration method produces the most reliable and least biased SSRT estimation (for further details and an exhaustive review see Verbruggen et al., 2019). Data were analyzed offline using custom-made MATLAB scripts (The MathWorks, Inc., Natick, MA, United States) estimating SSRT as described, and all statistical analyses were performed with STATISTICA (StatSoft STATISTICA 13, Tulsa, OK, United States). Mixed-design ANOVAs were used to investigate differences within and between groups. *Post hoc* analyses were conducted with Bonferroni test and the significance threshold was set at *p* < 0.05.

### Bayesian Statistics

The null hypothesis-testing analyses were complemented by their Bayesian implementations using JASP v 0.9.2 (Team, 2017). With Bayesian hypothesis testing, we could directly evaluate the relative strength of evidence for the null and alternative hypotheses, providing quantification of the degree to which the data support either hypothesis (Dienes, 2011; Wagenmakers et al., 2018). We used default priors in JASP for Bayesian *t*-tests (zero-centered Cauchy prior width, *r* = 0.707), Bayesian ANOVAs (*r* scale fixed effects = 0.5; *r* scale random effects = 1; *r* scale covariates = 0.354) and Bayesian correlations (stretched beta prior width, *r* = 1). Following the current standards, we report subscripts on Bayes factors (BFs) to refer to the models compared. Accordingly, the BF for the alternative relative to the null hypothesis is denoted BF_10_, while the BF for the null relative to the alternative hypothesis is denoted BF_01_. We interpreted and labeled the sizes of BFs according to the recommendations of Raftery (1995) as referred to by Jarosz and Jennifer (2014) and Kelter (2020) who suggested that, if the prior odds are assumed to be 1, an interpretation of BFs as anecdotal-to-positive evidence for the hypotheses is when BF values range from 1 to 3, and over 5 to 10 for moderate-to-strong evidence.

## Results

### Verification of the Correct Assumptions Underlying the Stop-Signal Task Data Collected

Firstly, we verified the correct assumptions of the independent race model (Verbruggen et al., 2019). In particular, we assessed by comparing whether the mean RT on unsuccessful Stop-trials (i.e., trials in which participants could not desist from performing an action even though a Stop-signal was presented) was shorter than the mean RT on Go trials (see Table 3 for descriptive SST data).

**TABLE 3:** Behavioral data.

	SARS-CoV-2		Fear-Face		Fear-Body	
			
SST	Negative	Neutral	Negative	Neutral	Negative	Neutral
Inhibition rate (%)	51.83 ± 6.03	51.51 ± 6.71	53.02 ± 5.97	52.70 ± 5.44	52.65 ± 8.41	51.92 ± 7.54
SSD (ms)	282.88 ± 117.08	275.17 ± 115.67	293.64 ± 113.93	289.08 ± 115.06	283.43 ± 121.12	276.84 ± 119.59
SSRT (ms)	233.45 ± 38.27	242.46 ± 35.12	227.05 ± 61.19	235.07 ± 52.68	234.49 ± 47.83	242.41 ± 46.06
Unsucc RT (ms)	479.61 ± 97.74	482.03 ± 101.91	490.91 ± 97.73	488.71 ± 97.35	481.74 ± 98.77	476.74 ± 96.52
Go RT (ms)	545.26 ± 128.39		562.84 ± 145.78		549.22 ± 136.03	
Correct Go (%)	98.85 ± 1.32		98.38 ± 2.16		98.28 ± 1.75	

*Descriptive performance of the stop-signal task (SST) is reported as means ± SD. In particular, inhibition rate, stop-signal delay (SSD), stop-signal reaction time (SSRT), unsuccessful reaction time (Unsucc RT), Go reaction time (Go RT), and correct Go responses are depicted in the table for each group.*

**TABLE 4:** Questionnaires data.

Group	STAI-Y2	HADS-anxiety	HADS-depression	BIS-11			
				Total score	Motoric impulsivity	Attentional impulsivity	Non-planning impulsivity
SARS-CoV-2	45.17 ± 9.11	8.00 ± 4.13	4.70 ± 3.72	64.37 ± 8.61	16.67 ± 3.24	21.23 ± 3.96	26.47 ± 4.17
Fear-Face	46.77 ± 8.15	7.00 ± 3.27	5.97 ± 3.20	62.67 ± 6.8	16.60 ± 3.09	19.57 ± 3.44	26.50 ± 3.66
Fear-Body	47.43 ± 9.55	6.97 ± 2.95	5.00 ± 3.13	64.70 ± 5.69	18.10 ± 2.72	20.90 ± 4.38	25.70 ± 3.62

*Scores are reported as mean ± SD.*

Subsequently, we made sure the staircase procedure was successful, ascertaining that the inhibition rate (i.e., percentage of stop performance when Stop-signal is presented) was approximately 50% for all stimuli during the task (SARS-CoV-2 group: Emotional = 51.83 ± 6.03%, Neutral = 51.51 ± 6.71%; Fear-Face group: Emotional = 53.02 ± 5.97%, Neutral = 52.70 ± 5.44%; Fear-Body group: Emotional = 52.65 ± 8.41%, Neutral = 51.92 ± 7.54%; see Table 3). To investigate differences across groups a 2 × 3 ANOVA on the percentage of the stop performance (i.e., inhibition rate) with Stimulus (Emotional/Neutral) as within-subject factor and Group (SARS-CoV-2/Fear-Face/Fear-Body) as between-subject factor was carried out. The analysis revealed that the inhibition rate did not differ between groups [*F*(2,87) = 0.242, *p* = 0.78, η*_*p*_*^2^ = 0.005], nor was it influenced by the emotional content of the Stimulus [*F*(1,87) = 2.631, *p* = 0.11, η*_*p*_*^2^ = 0.029]. Moreover, no Stimulus by Group interaction was found [*F*(2,87) = 0.237, *p* = 0.79, η*_*p*_*^2^ = 0.005; see Table 3 for descriptive SST data]. Furthermore, Bayesian ANOVA provided positive evidence in favor of the null hypothesis of no difference among groups (BF_01_ = 7.339). These results indicated that the percentage of the stop performance, when the Stop-signal is presented, was comparable both for the two stimuli and for all participants regardless of the group.

Similarly, we investigated the percentage of correct responses on Go-trials across groups using a 2 × 3 ANOVA with Go-responses (Correct/Incorrect) as within-subject factor and Group (SARS-CoV-2/Fear-Face/Fear-Body) as between-subject factor. The analysis revealed a main effect of Go-responses [*F*(1,87) = 66.7, *p* < 0.001, η*_*p*_*^2^ = 0.998], but no main effect of Group or Go-responses by Group interaction [*F*(2,87) = 8.81, *p* = 0.41, η*_*p*_*^2^ = 0.019], suggesting that all participants regardless of the group, had a similar performance in discriminating the direction of the arrow presented as the Go-signal. Follow-up simple paired *t*-tests [*t*(89) = 258.73, *p* < 0.001] revealed that correct Go-responses (98.5 ± 1.77%) were significantly higher than incorrect ones (1.49 ± 1.77%; see Table 3 for descriptive SST data), suggesting that the SST was correctly executed by the participants. In addition, Bayesian ANOVA provided positive evidence in favor of the null hypothesis of no difference among groups (BF_01_ = 3.845). Accordingly, Bayesian *t*-test showed positive evidence in favor of the alternative hypothesis of difference between correct versus incorrect responses (all BF_10_ > 30).

Finally, to assess sequential effects on RTs following Go-trials, a one-way ANOVA on the Go-RTs was performed. The analysis revealed no differences in RTs between groups [*F*(2,87) = 0.13, *p* = 0.87, η*_*p*_*^2^ = 0.003; see Table 3 for descriptive SST data]. Accordingly, Bayesian ANOVA provided positive evidence in favor of the null hypothesis of no difference among groups (BF_01_ = 9.021).

In conclusion, given these preliminary analyses, the SST data collected can be considered reliable and the assumption of correct inhibition rate has been verified. Thus, it is possible to reliably estimate the SSRT values (Verbruggen et al., 2019).

### Negative Emotional Content of Stimuli Enhances the Ability to Disrupt an Ongoing Action

**FIGURE 3:**
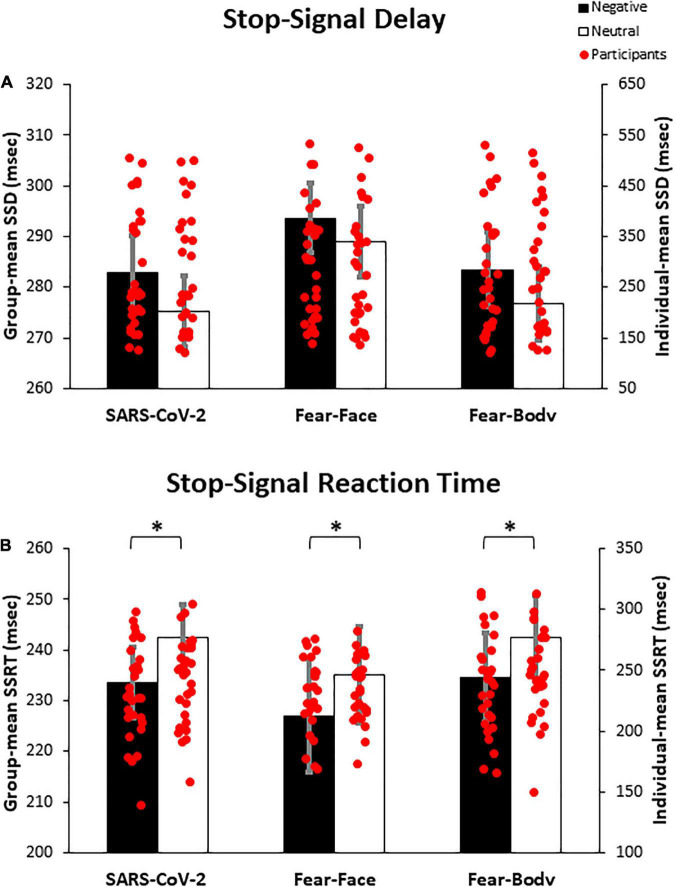
Bar graph of the experimental results. In **(A)** the graph shows the mean stop-signal delay (SSD), demonstrating that negative emotional content of stimuli influenced the participant’s action execution leading to a specific differentiation of SSD, given the successful staircase procedure. In **(B)** the graph shows the mean stop-signal reaction time (SSRT), demonstrating that participants showed a better inhibitory process when facing negative Stop-signals as compared to neutral ones, regardless of the group. Asterisks indicate significant comparisons (*p* < 0.05), and error bars represent SEM. Asterisks indicate significant comparisons.

Prior to the main analysis of the study, SSD data were analyzed using a 2 × 3 ANOVA with Stimulus (Emotional/Neutral) as within-subject factor and Group (SARS-CoV-2/Fear-Face/Fear-Body) as between-subject factor. The analysis revealed the main effect only of Stimulus [*F*(1,87) = 15.08, *p* < 0.001, η*_*p*_*^2^ = 0.14] and Bonferroni *post hoc* comparison showed significantly longer SSD for negative (286.65 ± 116.19 ms) than neutral Stop-signals stimuli (280.37 ± 115.64 ms; *p* < 0.001; Figure 3A). No other main effects or interactions were found to be significant (all *F* < 0.32; *p* = 0.72; η*_*p*_*^2^ < 0.007; see Table 3 for descriptive SST data). Moreover, Bayesian ANOVA provided positive evidence in favor of the null hypothesis of no difference among groups (BF_01_ = 6.934). As expected, the negative emotional content of stimuli influenced the participant’s actions execution leading to a specific differentiation of SSD that was properly adjusted given the successful staircase procedures.

Crucially, to verify the main hypothesis of the present study, SSRT data were analyzed using a 2 × 3 ANOVA with Stimulus (Emotional/Neutral) as within-subject factor and Group (SARS-CoV-2/Fear-Face/Fear-Body) as between-subject factor. Results showed the main effect only of Stimulus [*F*(1,87) = 14.999, *p* < 0.001, η*_*p*_*^2^ = 0.147]. Bonferroni *post hoc* comparisons showed that SSRTs were significantly lower (*p* < 0.001) for negative stimuli (231.67 ± 49.53 ms) with respect to neutral ones (239.98 ± 44.83 ms). No other main effects or interaction were found to be significant (all *F* < 0.236; *p* = 0.79; η*_*p*_*^2^ < 0.006; see Table 3 for descriptive SST data). Furthermore, Bayesian ANOVA provided positive evidence in favor of the null hypothesis of no difference among groups (BF_01_ = 9.887). To further investigate the effect of emotion in the SSRT, follow-up simple paired *t*-tests revealed that SSRT was significantly reduced for the negative emotion condition compared to its neutral counterpart in the SARS-CoV-2 [*t*(29) = −2.41, *p* = 0.02], in the Fear-Face group [*t*(29) = −2.18, *p* = 0.03] and in the Fear-Body group [*t*(29) = −2.11, *p* = 0.04; see Figure 3B]. Accordingly, Bayesian *t*-test showed anecdotal evidence in favor of the alternative hypothesis of difference between emotion versus neutral condition (1.332 ≤ BF_10_ ≤ 2.294).

Finally, these results showed that participants were more capable in inhibiting responses with fearful Stop signals compared to neutral ones. Crucially, our results additionally demonstrated that the COVID-19 stimulus impacts the inhibitory process similarly to observing a fearful face or a fearful body.

### Non-planned Impulsivity, but Not Anxiety, Predicts Correct Inhibition for Negative Stimuli

To explore the relations between the better reactive action inhibition when facing negative stimuli and personality traits, correlation and regression analyses were performed. An index representing the inhibition for negative stimuli (i.e., SSRT of the negative stimulus minus the SSRT of the neutral stimulus) was considered as the dependent variable in a stepwise regression model, whereas scores for the STAI-Y2 and BIS-11 subscales were entered as predictors. The regression model was not found to be significant [*R*^2^ = 0.063; *F*(4,85) = 1.448; *p* = 0.22]. However, after the removal of three statistical outliers with residual >2 sigma which were present in the data set, the regression model resulted significant [*R*^2^ = 0.118; *F*(4,82) = 2.744; *p* = 0.03].

**FIGURE 4:**
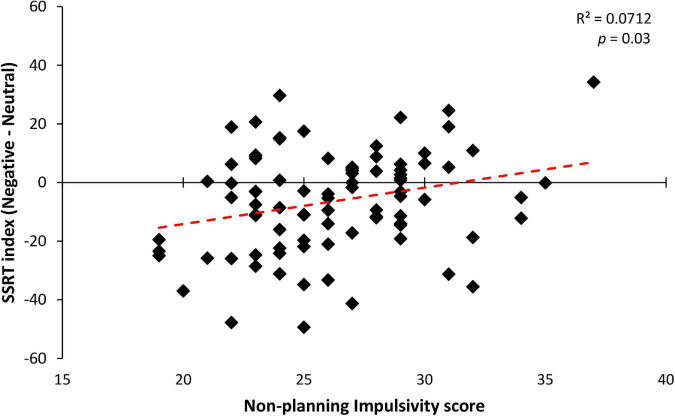
Significant correlation between non-planning impulsivity score (a subscale of the BIS-11 questionnaire) and SSRT index (calculated as Negative minus Neutral SSRT) in the three groups. Across participants, a significant linear relationship was observed, indicating that participants with higher levels of non-planned impulsivity showed a lower inhibitory performance improvement during the negative emotional stop conditions, with respect to their neutral counterparts.

Among the predictors, only the non-planning impulsivity component held a significant positive correlation with the SSRT index [*b* = 1.192; *t*(82) = 2.321; *p* = 0.02; see Figure 4]. Accordingly, Bayesian regression showed evidence in favor of the alternative hypothesis that non-planned impulsivity predicts correct reactive inhibition for negative stimuli (BF_10_ = 3.684). This result suggests that higher levels of non-planned impulsivity, which is defined as orientation toward the present rather than to the future (e.g., present-moment focus without regard for future consequences) (Dunne et al., 2019), lead to an advantage for emotional negative stimuli compared to neutral ones (see Table 4 for further details). Taken together these findings suggest that only impulsivity influences the participants’ ability to inhibit their actions. Specifically, only non-planning impulsivity correlated with the stopping advantage for negative stimuli, demonstrating that participants with lower self-control were less facilitated in their ability to suppress an ongoing action when presented with a negative Stop stimulus, resulting in a lack of emotional facilitation for inhibitory performance.

## Discussion

The processing of emotion-laden information, such as threat, is fast and prioritized. Indeed, negative stimuli have been found to rapidly suppress cortical or corticospinal excitability (Borgomaneri et al., 2015b,c, 2017, 2020b,^2021^; Vicario et al., 2017; Battaglia et al., 2021), which has been interpreted as a freezing-like inhibitory modulation of the primary motor cortex (Borgomaneri et al., 2015a). In line with this negative advantage effect, several studies have tried to disclose the impact of fearful stimuli over one of the fundamental executive human capabilities, namely the ability to inhibit an inappropriate action (Bari and Robbins, 2013; Mirabella, 2014). The principle has been applied to develop preclinical models of behaviors including depression, anxiety, fear, memory, and learning, among others (Tanaka et al., 2011, 2012; Palotai et al., 2014; Tanaka and Telegdy, 2014). The SST is designed to provide a sensitive measure of the time taken by the brain to inhibit or suppress inappropriate motor responses (Logan et al., 1997; Matzke et al., 2018). Most of the existing studies have employed SST using emotional stimuli and have demonstrated that the presentation of an emotional image before the Go stimulus generally interferes with our ability to stop an action (Verbruggen and De Houwer, 2007; Kalanthroff et al., 2013; Rebetez et al., 2015), while when the emotional stimulus is presented as the stop, a facilitatory effect has been generally reported (Pessoa et al., 2012; Senderecka, 2016, 2018), but see the modulatory role of task, as in Mancini et al. (2022). However, Pessoa et al. (2012) demonstrated that it is the intensity of the stimulus that may play a crucial role (i.e., dual competition framework), by showing that the presentation of low-threat fearful facial stimuli as stops facilitates action suppression, while the presentation of a high-threat stimulus, such as a fear conditioned tone, disrupted such ability. However, from these data it was not possible to disambiguate whether it was the intensity or the intrinsic negative nature of the stimuli that played a role in differently affecting motor control capabilities. Here, we aimed at further investigating this dual competition framework by presenting as stop in an SST, a fearful face/body or a SARS-CoV-2 image, which is an intrinsically neutral image that, however, through vicarious fear-learning (Olsson and Phelps, 2007; Debiec and Olsson, 2017) acquired through the massive media exposure to the health threats of the virus, was fear conditioned (Cinelli et al., 2020; Malecki et al., 2020). Indeed, similarly, to classical fear conditioning, vicarious fear-learning is a successful mechanism used for studying the transmission of threat information without directly obtaining the unconditioned stimulus (US), thus directly experiencing the painful stimulation. By taking advantage of vicarious fear-learning, our results showed that our three matched negative stimuli similarly affect our ability to stop our action, by reducing the SSRT and thus facilitating action control. Therefore, we can confirm Pessoa et al.’s (2012) findings by demonstrating that negative stimuli, although not intrinsically negative, improved response inhibition compared to neutral ones.

Important methodological differences between our study and Pessoa et al.’s (2012) should be mentioned. Indeed, Pessoa et al. (2012) conditioned a tone using a classical fear conditioning procedure, namely by delivering an electric annoying shock as an US, while in our case the SARS-CoV-2 image has become negative through a vicarious fear-learning process that did not directly involve physical pain. Despite this difference, our vicarious conditioned stimulus (i.e., the SARS-CoV-2 image) was associated with a fear response, and clearly influenced reactive action inhibition. It is possible to speculate that this fear for the COVID-19 image was associated with the fear of being infected by the virus. On top of this, it is also important to consider personality traits, such as anxiety and impulsivity, which might influence the ability to promptly stop an ongoing action when necessary. In fact, our results showed that the advantage in stopping provided by negative stimuli is linked to non-planning impulsivity, which can be thought of as present-moment focus without regard for future consequences (Dunne et al., 2019). More specifically, the higher the non-planning impulsivity score, the lower the advantage provided by negative stimuli. This result would suggest that impulsivity is an important factor in determining the ability to interrupt an ongoing action, even in the face of a negative scenario diverting participants from encoding emotion. A potential limitation of this study is the lack of measures of proactive inhibition. This aspect represents an important issue that future studies should investigate since it has become increasingly clear that specific patterns of reactive and proactive inhibitory control impairments shape the phenotypes of several psychiatric and neurological disorders characterized by poor urge control (for a review see Mirabella, 2021). Moreover, it has been reported that a crucial detail seems to be the role of emotional stimuli in task instruction. Indeed, in most of the existing studies, the valence of the stop stimuli was irrelevant to the required response. However, Mancini et al. (2022), using a Go/No-Go task, showed that fearful facial expressions improve inhibitory control (measured as the rate of commission error) with respect to happy (and not neutral) expressions but only when relevant to participants goals, i.e., when participants have to refrain from moving at the presentation of an emotional stimulus. These results are in line with findings that suggest how fearful emotional stimuli increase the RTs and the rate of commission errors with respect to happy facial expressions (Mirabella, 2018; Mancini et al., 2020). Differently, if they have to stop according to the actors’ gender, emotional stimuli had the same effect as neural facial expressions. Thus, task instruction may play an important role in modulating action control capabilities in an emotional context and future studies will be necessary to address this important point. Moreover, an intriguing possibility for future studies is to validate the neutrality of a particular virus images before they become fear conditioned (i.e., the Monkeypox virus) or using another biological entity (such as an image of cell) as control condition.

Finally, future studies will aim to investigate the neural basis of the integration between emotion and action control. Several areas in the prefrontal cortex have been associated with the mechanisms underlying inhibitory control, with a network including the inferior frontal gyrus (IFG), primary motor area (Mattia et al., 2012), pre-motor area (Cattaneo and Parmigiani, 2021), pre-supplementary motor area (pre-SMA; see Wessel and Aron, 2017; Zhang et al., 2017; Borgomaneri et al., 2020a for a comprehensive meta-analysis), posterior parietal cortex (Convento et al., 2014), and basal ganglia (Mallet et al., 2016). Indeed, several studies have attempted to disclose the crucial nodes involved in action control (Wessel et al., 2013; Mirabella et al., 2020) or in the control of fear responses (Borgomaneri et al., 2020b) by employing non-invasive brain stimulation techniques (NIBS) (Borgomaneri et al., 2020b; Battaglia et al., 2021). Recently, there has been a growing interest in the use of NIBS to selectively manipulate the activations of selective brain regions of the action inhibition network (AIN) to investigate their specific contribution to many processes underlying action control (i.e., inhibition, selection, competition, and switching of actions). Besides, disclosing the specific and critical role of the different components of the AIN represents a crucial challenge to pave the way for designing novel NIBS therapeutic interventions aimed at enhancing the ability to improve cognitive control and inhibit potentially dangerous actions. Crucially, however, none of the existing NIBS studies investigated the neural network at play when emotional stimuli are presented during an SST. Therefore, the specific role of different prefrontal regions in motor control is still a matter of debate. Interestingly, it remains obscure whether the same neural circuit is involved in reactive action inhibition or whether distinct inhibitory neural processes are at play when emotional information is presented. This finding may pave the way for future therapeutic strategies based on the administration of NIBS to modulate the impact of emotional stimuli on our cognitive abilities.

## Conclusion

Herein we provided evidence that emotionally negative stimuli, although not intrinsically negative, are able to facilitate our action control abilities. Our data demonstrate the power of vicarious fear learning in influencing our behavior and provide a demonstration that the image of the SARS-CoV-2, through massive media exposure processing, has become a potentially negative stimulus even though it only represents the virus and not the disease *per se*. Future studies will aim to investigate whether, with the passage of time and the reduction in the mortality of the disease, the SARS-CoV-2 image will extinguish or else reconsolidate its aversive memory.

## Data Availability Statement

The datasets collected and analyzed during the current study are available from the corresponding authors on reasonable request, due to concerns about privacy, health status (i.e., SARS-CoV-2 diagnosis), and confidentiality of our participants.

## Ethics Statement

The studies involving human participants were reviewed and approved by the Ethics Committee of the Department of Psychology of the University of Bologna. The patients/participants provided their written informed consent to participate in this study.

## Author Contributions

SBa and SBo conceived and designed the study concept and wrote the manuscript. PC developed the local-only version of a classical stop-signal task in JavaScript, while SBa developed the jsPsych-version of the stop-signal task customized for the present study. CDF and CN performed the SST data collection, questionnaire scoring, and analysis. SBa performed the SST data analysis and designed the figures and tables. All authors approved the final version of the manuscript for submission.

## Conflict of Interest

The authors declare that the research was conducted in the absence of any commercial or financial relationships that could be construed as a potential conflict of interest.

## Publisher’s Note

All claims expressed in this article are solely those of the authors and do not necessarily represent those of their affiliated organizations, or those of the publisher, the editors and the reviewers. Any product that may be evaluated in this article, or claim that may be made by its manufacturer, is not guaranteed or endorsed by the publisher.

## Footnotes

1
https://www.ecma-international.org/publications/standards/Ecma-006.htm

2
https://www.jetbrains.com/idea/

3
https://firebase.google.com/

4
https://github.com/

5
https://www.php.net/

6
https://www.google.com/forms/about/

## References

[B1] Aron A. R. (2011). From reactive to proactive and selective control: developing a richer model for stopping inappropriate responses. *Biol. Psychiatry* 69 e55–e68. 10.1016/j.biopsych.2010.07.024 20932513 PMC3039712

[B2] Avila C. Parcet M. A. (2001). Personality and inhibitory deficits in the stop-signal task: the mediating role of Gray’s anxiety and impulsivity. *Pers Indiv. Dif.* 31 975–986. 10.1016/S0191-8869(00)00199-9

[B3] Balbuena L. Monaro M. (2021). Fear of infection and the common good: COVID-19 and the first italian lockdown. *Int. J. Environ. Res. Public Health* 18:21. 10.3390/ijerph182111341 34769858 PMC8583192

[B4] Band G. P. H. van der Molen M. W. Logan G. D. (2003). Horse-race model simulations of the stop-signal procedure. *Acta Psychol. (Amst).* 112 105–142. 10.1016/S0001-6918(02)00079-3 12521663

[B5] Bari A. Robbins T. W. (2013). Inhibition and impulsivity: behavioral and neural basis of response control. *Prog. Neurobiol.* 108 44–79. 10.1016/j.pneurobio.2013.06.005 23856628

[B6] Battaglia S. Serio G. Scarpazza C. D’Ausilio A. Borgomaneri S. (2021). Frozen in (e)motion: how reactive motor inhibition is influenced by the emotional content of stimuli in healthy and psychiatric populations. *Behav. Res. Ther.* 146:103963. 10.1016/j.brat.2021.103963 34530318

[B7] Borgomaneri S. Gazzola V. Avenanti A. (2015a). Transcranial magnetic stimulation reveals two functionally distinct stages of motor cortex involvement during perception of emotional body language. *Brain Struct. Funct.* 220 2765–2781. 10.1007/s00429-014-0825-6 25023734 PMC4549387

[B8] Borgomaneri S. Vitale F. Avenanti A. (2015b). Early changes in corticospinal excitability when seeing fearful body expressions. *Sci. Rep.* 5:14122. 10.1038/srep14122 26388400 PMC4585670

[B9] Borgomaneri S. Vitale F. Gazzola V. Avenanti A. (2015c). Seeing fearful body language rapidly freezes the observer’s motor cortex. *Cortex* 65 232–245. 10.1016/j.cortex.2015.01.014 25835523

[B10] Borgomaneri S. Serio G. Battaglia S. (2020a). Please, don’t do it! Fifteen years of progress of non-invasive brain stimulation in action inhibition. *Cortex* 132 404–422. 10.1016/j.cortex.2020.09.002 33045520

[B11] Borgomaneri S. Vitale F. Avenanti A. (2020b). Early motor reactivity to observed human body postures is affected by body expression, not gender. *Neuropsychologia* 146:107541. 10.1016/j.neuropsychologia.2020.107541 32593723

[B12] Borgomaneri S. Vitale F. Avenanti A. (2017). Behavioral inhibition system sensitivity enhances motor cortex suppression when watching fearful body expressions. *Brain Struct. Funct.* 222 3267–3282. 10.1007/s00429-017-1403-5 28357586

[B13] Borgomaneri S. Vitale F. Battaglia S. Avenanti A. (2021). Early right motor cortex response to happy and fearful facial expressions: a TMS motor-evoked potential study. *Brain Sci.* 11:1203. 10.3390/brainsci11091203 34573224 PMC8471632

[B14] Cai Y. Li S. Liu J. Li D. Feng Z. Wang Q. (2015). The role of the frontal and parietal cortex in proactive and reactive inhibitory control: a transcranial direct current stimulation study. *J. Cogn. Neurosci.* 28 177–186. 10.1162/jocn_a_00888 26439269

[B15] Cattaneo L. Parmigiani S. (2021). Stimulation of different sectors of the human dorsal premotor cortex induces a shift from reactive to predictive action strategies and changes in motor inhibition: a dense transcranial magnetic stimulation (tms) mapping study. *Brain Sci.* 11:5. 10.3390/brainsci11050534 33923217 PMC8146001

[B16] Choi J. M. Cho Y. S. (2020). Beneficial effect of task-irrelevant threat on response inhibition. *Acta Psychol. (Amst).* 202:102980. 10.1016/j.actpsy.2019.102980 31785576

[B17] Cinelli M. Quattrociocchi W. Galeazzi A. Valensise C. M. Brugnoli E. Schmidt A. L. (2020). The COVID-19 social media infodemic. *Sci. Rep.* 10:16598. 10.1038/s41598-020-73510-5 33024152 PMC7538912

[B18] Convento S. Bolognini N. Fusaro M. Lollo F. Vallar G. (2014). Neuromodulation of parietal and motor activity affects motor planning and execution. *Cortex* 57 51–59. 10.1016/j.cortex.2014.03.006 24769545

[B19] D’Agostino A. Demartini B. Cavallotti S. Gambini O. (2020). Mental health services in Italy during the COVID-19 outbreak. *Lancet Psychiatry* 7 385–387. 10.1016/S2215-0366(20)30133-4 32353266 PMC7185925

[B20] De Leeuw J. R. (2015). jsPsych: a javascript library for creating behavioral experiments in a web browser. *Behav. Res. Methods* 47 1–12. 10.3758/s13428-014-0458-y 24683129

[B21] De Leeuw J. R. Motz B. A. (2016). Psychophysics in a web browser? Comparing response times collected with javascript and psychophysics toolbox in a visual search task. *Behav. Res. Methods* 48 1–12. 10.3758/s13428-015-0567-2 25761390

[B22] Debiec J. Olsson A. (2017). Social fear learning: from animal models to human function. *Trends Cogn. Sci.* 21 546–555. 10.1016/j.tics.2017.04.010 28545935 PMC5507357

[B23] Dienes Z. (2011). Bayesian versus orthodox statistics: which side are you on? *Perspect. Psychol. Sci. J. Assoc. Psychol. Sci.* 6 274–290. 10.1177/1745691611406920 26168518

[B24] Dunne E. Cook R. Ennis N. (2019). Non-planning impulsivity but not behavioral impulsivity is associated with HIV medication non-adherence. *AIDS Behav.* 23 1297–1305. 10.1007/s10461-018-2278-z 30264205 PMC6437004

[B25] Ekman P. Friesen W. V. (1976). *Pictures of Facial Affect.* Palo Alto, CA: Consulting Psychologists Press.

[B26] Faul F. Erdfelder E. Lang A.-G. Buchner A. (2007). G*power 3: a flexible statistical power analysis program for the social, behavioral, and biomedical sciences. *Behav. Res. Methods* 39 175–191. 10.3758/BF03193146 17695343

[B27] Herbert C. Sütterlin S. (2011). Response inhibition and memory retrieval of emotional target words: evidence from an emotional stop-signal task. *J. Behav. Brain Sci.* 1 153–159. 10.4236/jbbs.2011.13020

[B28] Hilbig B. E. (2016). Reaction time effects in lab- versus web-based research: experimental evidence. *Behav. Res. Methods* 48 1718–1724. 10.3758/s13428-015-0678-9 26542972

[B29] Hilt P. M. Cardellicchio P. (2020). Attentional bias on motor control: is motor inhibition influenced by attentional reorienting? *Psychol. Res.* 84 276–284. 10.1007/s00426-018-0998-3 29520490

[B30] Holmes E. A. O’Connor R. C. Perry V. H. Tracey I. Wessely S. Arseneault L. (2020). Multidisciplinary research priorities for the COVID-19 pandemic: a call for action for mental health science. *Lancet Psychiatry* 7 547–560. 10.1016/S2215-0366(20)30168-1 32304649 PMC7159850

[B31] Hsieh M. Lu H. Chen L. Liu C. Hsu S. Cheng C. (2022). Cancellation but not restraint ability is modulated by trait anxiety?: an event-related potential and oscillation study using go-nogo and stop-signal tasks. *J. Affect. Dis.* 299 188–195. 10.1016/j.jad.2021.11.066 34863714

[B32] Jarosz A. F. Wiley J. (2014). What are the odds? A practical guide to computing and reporting Bayes factors. *J. Probl. Solving* 7:2. 10.7771/1932-6246.1167 29356800

[B33] Kalanthroff E. Cohen N. Henik A. (2013). Stop feeling: inhibition of emotional interference following stop-signal trials. *Front. Hum. Neurosci.* 7:1–7. 10.3389/fnhum.2013.00078 23503817 PMC3596782

[B34] Kelter R. (2020). Bayesian alternatives to null hypothesis significance testing in biomedical research: a non-technical introduction to bayesian inference with JASP. *BMC Med. Res. Methodol.* 20:142. 10.1186/s12874-020-00980-6 32503439 PMC7275319

[B35] Kisely S. Warren N. McMahon L. Dalais C. Henry I. Siskind D. (2020). Occurrence, prevention, and management of the psychological effects of emerging virus outbreaks on healthcare workers: rapid review and meta-analysis. *BMJ* 369:m1642. 10.1136/bmj.m1642 32371466 PMC7199468

[B36] Lai J. Ma S. Wang Y. Cai Z. Hu J. Wei N. (2020). Factors associated with mental health outcomes among health care workers exposed to coronavirus disease 2019. *JAMA Netw Open.* 3:e203976. 10.1001/jamanetworkopen.2020.3976 32202646 PMC7090843

[B37] Lappin J. S. Eriksen C. W. (1966). Use of a delayed signal to stop a visual reaction-time response. *J. Exp. Psychol.* 72 805–811. 10.1037/h0021266

[B38] Lee A. M. Wong J. G. W. S. McAlonan G. M. Cheung V. Cheung C. Sham P. C. (2007). Stress and psychological distress among SARS survivors 1 year after the outbreak. *Can. J. Psychiatry* 52 233–240. 10.1177/070674370705200405 17500304

[B39] Legrand A. C. Price M. (2020). Emotionally valenced stimuli impact response inhibition in those with substance use disorder and co-occurring anxiety and depression symptoms. *J. Affect. Disord.* 266 639–645. 10.1016/j.jad.2020.02.008 32056940 PMC7105387

[B40] Lei L. Huang X. Zhang S. Yang J. Yang L. Xu M. (2020). Comparison of prevalence and associated factors of anxiety and depression among people affected by versus people unaffected by quarantine during the COVID-19 epidemic in Southwestern China. *Med. Sci. Monit.* 26:e924609. 10.12659/MSM.924609 32335579 PMC7199435

[B41] Lisi M. P. Scattolin M. Fusaro M. Aglioti S. M. (2021). A bayesian approach to reveal the key role of mask wearing in modulating projected interpersonal distance during the first COVID-19 outbreak. *PLoS One* 16:e0255598. 10.1371/journal.pone.0255598 34375361 PMC8354471

[B42] Logan G. D. (1994). “On the ability to inhibit thought and action: a users’ guide to the stop signal paradigm,” in *Inhib Process Attention, Mem Lang*, eds Dagenbach D. Carr T. H. 189–239.

[B43] Logan G. D. Cowan W. B. (1984). On the ability to inhibit thought and action: a theory of an act of control. *Psychol. Rev.* 91 295–327. 10.1037/0033-295X.91.3.295 24490789

[B44] Logan G. D. Schachar R. J. Tannoc R. (1997). Impulsivity and inhibitory control. *Psychol. Sci.* 8 60–64. 10.1111/j.1467-9280.1997.tb00545.x

[B45] Logan G. D. Van Zandt T. Verbruggen F. Wagenmakers E. J. (2014). On the ability to inhibit thought and action: general and special theories of an act of control. *Psychol. Rev.* 121 66–95. 10.1037/a0035230 24490789

[B46] Malecki K. M. C. Keating J. A. Safdar N. (2020). Crisis communication and public perception of COVID-19 risk in the era of social media. *Clin. Infect. Dis.* 2020:ciaa758. 10.1093/cid/ciaa758 32544242 PMC7337650

[B47] Mallet N. Schmidt R. Leventhal D. Chen F. Amer N. Boraud T. (2016). Arkypallidal cells send a Stop signal to Striatum. *Neuron* 89 308–316. 10.1016/j.neuron.2015.12.017 26777273 PMC4871723

[B48] Mancini C. Falciati L. Maioli C. Mirabella G. (2020). Threatening facial expressions impact goal-directed actions only if task-relevant. *Brain Sci.* 10:11. 10.3390/brainsci10110794 33138170 PMC7694135

[B49] Mancini C. Falciati L. Maioli C. Mirabella G. (2022). Happy facial expressions impair inhibitory control with respect to fearful facial expressions but only when task-relevant. *Emotion* 22 142–152. 10.1037/emo0001058 34968143

[B50] Martos D. Tuka B. Tanaka M. Vécsei L. Telegdy G. (2022). Memory enhancement with kynurenic acid and its mechanisms in neurotransmission. *Biomedicines* 10:2022. 10.3390/biomedicines10040849 35453599 PMC9027307

[B51] Mattia M. Spadacenta S. Pavone L. Quarato P. Esposito V. Sparano A. (2012). Stop-event-related potentials from intracranial electrodes reveal a key role of premotor and motor cortices in stopping ongoing movements. *Front. Neuroeng.* 5:1–13. 10.3389/fneng.2012.00012 22754525 PMC3386527

[B52] Matzke D. Verbruggen F. Logan G. D. (2018). *The Stop-Signal Paradigm*. *Stevens’ Handbook of Experimental Psychology and Cognitive Neuroscience*, Wiley, 5th Edition. 1–45. 10.1002/9781119170174.epcn510

[B53] Mirabella G. (2014). Should I stay or should I go? Conceptual underpinnings of goal-directed actions. *Front. Syst. Neurosci.* 8:1–21. 10.3389/fnsys.2014.00206 25404898 PMC4217496

[B54] Mirabella G. (2018). The weight of emotions in decision-making: How fearful and happy facial stimuli modulate action readiness of goal-directed actions. *Front. Psychol.* 9:1–8. 10.3389/fpsyg.2018.01334 30116211 PMC6083043

[B55] Mirabella G. (2021). Inhibitory control and impulsive responses in neurodevelopmental disorders. *Dev. Med. Child. Neurol.* 63 520–526. 10.1111/dmcn.14778 33340369

[B56] Mirabella G. Upadhyay N. Mancini C. Giannì C. Panunzi S. Petsas N. (2020). Loss in grey matter in a small network of brain areas underpins poor reactive inhibition in obsessive-compulsive disorder patients. *Psychiatry Res. Neur.* 297:111044. 10.1016/j.pscychresns.2020.111044 32078965

[B57] Neo P. S. H. Thurlow J. K. McNaughton N. (2011). Stopping, goal-conflict, trait anxiety and frontal rhythmic power in the stop-signal task. *Cogn. Affect. Behav. Neurosci.* 11 485–493. 10.3758/s13415-011-0046-x 21647572

[B58] Olsson A. Phelps E. A. (2007). Social learning of fear. *Nat. Neurosci.* 10 1095–1102. 10.1038/nn1968 17726475

[B59] Palotai M. Telegdy G. Tanaka M. Bagosi Z. Jászberényi M. (2014). Neuropeptide AF induces anxiety-like and antidepressant-like behavior in mice. *Behav. Brain Res.* 274 264–269. 10.1016/j.bbr.2014.08.007 25116251

[B60] Patterson T. K. Lenartowicz A. Berkman E. T. Ji D. Poldrack R. A. Knowlton B. J. (2016). Putting the brakes on the brakes: negative emotion disrupts cognitive control network functioning and alters subsequent stopping ability. *Exp. Brain Res.* 234 3107–3118. 10.1007/s00221-016-4709-2 27349996 PMC5073018

[B61] Patton J. H. Stanford M. S. Barratt E. S. (1995). Factor structure of the barratt impulsiveness scale. *J Clin. Psychol.* 51, 768–774. 10.1002/1097-4679(199511)51:6<768::AID-JCLP2270510607>3.0.CO;2-1 8778124

[B62] Pawliczek C. M. Derntl B. Kellermann T. Kohn N. Gur R. C. Habel U. (2013). Inhibitory control and trait aggression: neural and behavioral insights using the emotional stop signal task. *Neuroimage* 79 264–274. 10.1016/j.neuroimage.2013.04.104 23660028

[B63] Pessoa L. (2009). How do emotion and motivation direct executive control? *Trends Cogn. Sci.* 13 160–166. 10.1016/j.tics.2009.01.006 19285913 PMC2773442

[B64] Pessoa L. Padmala S. Kenzer A. Bauer A. (2012). Interactions between cognition and emotion during response inhibition. *Emotion* 12 192–197. 10.1037/a0024109 21787074 PMC3208031

[B65] Pfefferbaum B. Schonfeld D. Flynn B. W. Norwood A. E. Dodgen D. Kaul R. E. (2012). The integration of mental and behavioral health into disaster preparedness. *Response Recovery* 6 60–66. 10.1001/dmp.2012.1 22490938

[B66] Pinet S. Zielinski C. Mathôt S. Dufau S. Alario F. X. Longcamp M. (2017). Measuring sequences of keystrokes with jsPsych: reliability of response times and interkeystroke intervals. *Behav. Res. Methods* 49 1163–1176. 10.3758/s13428-016-0776-3 27412730

[B67] Quadros S. Garg S. Ranjan R. Vijayasarathi G. Mamun M. A. (2021). Fear of COVID 19 infection across different cohorts: a scoping review. *Front. Psychiatry* 12:708430. 10.3389/fpsyt.2021.708430 34557117 PMC8453018

[B68] Raftery A. E. (1995). Bayesian model selection in social research. *Sociol. Methodol.* 25 111–196. 10.2307/271063

[B69] Rebetez M. M. L. Rochat L. Billieux J. Gay P. Van der Linden M. (2015). Do emotional stimuli interfere with two distinct components of inhibition? *Cogn. Emot.* 29 559–567. 10.1080/02699931.2014.922054 24885111

[B70] Reimers S. Stewart N. (2015). Presentation and response timing accuracy in adobe flash and HTML5/javascript web experiments. *Behav. Res. Methods* 47 309–327. 10.3758/s13428-014-0471-1 24903687 PMC4427652

[B71] Reissman D. B. Watson P. J. Klomp R. W. Tanielian T. L. Prior S. D. (2006). Pandemic influenza preparedness: adaptive responses to an evolving challenge. *J. Homel. Secur. Emerg. Manage.* 3 1–27. 10.2202/1547-7355.1233

[B72] Sagaspe P. Schwartz S. Vuilleumier P. (2011). Fear and stop: a role for the amygdala in motor inhibition by emotional signals. *Neuroimage* 55 1825–1835. 10.1016/j.neuroimage.2011.01.027 21272655

[B73] Senderecka M. (2016). Threatening visual stimuli influence response inhibition and error monitoring: an event-related potential study. *Biol. Psychol.* 113 24–36. 10.1016/j.biopsycho.2015.11.003 26599814

[B74] Senderecka M. (2018). Emotional enhancement of error detection—the role of perceptual processing and inhibition monitoring in failed auditory stop trials. *Cogn. Affect. Behav. Neurosci.* 18 1–20. 10.3758/s13415-017-0546-4 29076064 PMC5823965

[B75] Shi J. Gao Y. Zhao L. Li Y. Yan M. Niu M. M. (2020). Prevalence of delirium, depression, anxiety, and post-traumatic stress disorder among COVID-19 patients: protocol for a living systematic review. *Syst. Rev.* 9:258. 10.1186/s13643-020-01507-2 33158456 PMC7646715

[B76] Sohrabi C. Alsafi Z. O’Neill N. Khan M. Kerwan A. Al-Jabir A. (2020). World health organization declares global emergency: a review of the 2019 novel coronavirus (COVID-19). *Int. J. Surg.* 76 71–76. 10.1016/j.ijsu.2020.02.034 32112977 PMC7105032

[B77] Spekker E. Tanaka M. Szabó Á Vécsei L. (2021). Neurogenic inflammation: the participant in migraine and recent advancements in translational research. *Biomedicines* 10:1. 10.3390/biomedicines10010076 35052756 PMC8773152

[B78] Spielberger C. Gorsuch R. Lushene R. Vagg P. Jacobs G. (1983). *Manual for the State-Trait Anxiety Inventory.* Palo Alto, CA: Consulting Psychologists Press, Inc.

[B79] Stockdale L. A. Morrison R. G. Silton R. L. (2020). The influence of stimulus valence on perceptual processing of facial expressions and subsequent response inhibition. *Psychophysiology* 57 1–13. 10.1111/psyp.13467 31454096

[B80] Tanaka M. Telegdy G. (2014). Neurotransmissions of antidepressant-like effects of neuromedin U-23 in mice. *Behav. Brain Res.* 259 196–199. 10.1016/j.bbr.2013.11.005 24239690

[B81] Tanaka M. Kádár K. Tóth G. Telegdy G. (2011). Antidepressant-like effects of urocortin 3 fragments. *Brain Res. Bull.* 84 414–418. 10.1016/j.brainresbull.2011.01.016 21295118

[B82] Tanaka M. Schally A. V. Telegdy G. (2012). Neurotransmission of the antidepressant-like effects of the growth hormone-releasing hormone antagonist MZ-4-71. *Behav. Brain Res.* 228 388–391. 10.1016/j.bbr.2011.12.022 22197299

[B83] Tanaka M. Spekker E. Szabó Á Polyák H. Vécsei L. (2022). Modelling the neurodevelopmental pathogenesis in neuropsychiatric disorders. bioactive kynurenines and their analogues as neuroprotective agents-in celebration of 80th birthday of professor peter riederer. *J. Neural. Transm.* 129, 627–642. 10.1007/s00702-022-02513-5 35624406

[B84] Tanaka M. Tóth F. Polyák H. Szabó Á Mándi Y. Vécsei L. (2021). Immune influencers in action: metabolites and enzymes of the tryptophan-kynurenine metabolic pathway. *Biomedicines* 9:7. 10.3390/biomedicines9070734 34202246 PMC8301407

[B85] Taylor S. Landry C. A. Rachor G. S. Paluszek M. M. Asmundson G. J. G. (2020). Fear and avoidance of healthcare workers: an important, under-recognized form of stigmatization during the COVID-19 pandemic. *J. Anxiety Disord.* 75:102289. 10.1016/j.janxdis.2020.102289 32853884 PMC7434636

[B86] Team J. (2017). *p. Jasp (Version 0.8.5) [Computer Software].*

[B87] Veer I. M. Riepenhausen A. Zerban M. Wackerhagen C. Puhlmann L. M. C. Engen H. (2021). Psycho-social factors associated with mental resilience in the Corona lockdown. *Transl. Psychiatry* 11:67. 10.1038/s41398-020-01150-4 33479211 PMC7817958

[B88] Verbruggen F. De Houwer J. (2007). Do emotional stimuli interfere with response inhibition? Evidence from the stop signal paradigm. *Cogn. Emot.* 21 391–403. 10.1080/02699930600625081 34530318

[B89] Verbruggen F. Aron A. R. Band G. P. H. Beste C. Bissett P. G. Brockett A. T. (2019). A consensus guide to capturing the ability to inhibit actions and impulsive behaviors in the stop-signal task. *Elife* 8 1–26. 10.7554/eLife.46323.027 31033438 PMC6533084

[B90] Vicario C. M. Rafal R. D. Borgomaneri S. Paracampo R. Kritikos A. Avenanti A. (2017). Pictures of disgusting foods and disgusted facial expressions suppress the tongue motor cortex. *Soc. Cogn. Affect. Neurosci.* 12 352–362. 10.1093/scan/nsw129 27614770 PMC5390717

[B91] Vince M. A. (1948). The intermittency of control movements and the psychological refrectory period. *J. Psychol.* 38 149–157. 10.1111/j.2044-8295.1948.tb01150.x 18913658

[B92] Vindegaard N. Benros M. E. (2020). COVID-19 pandemic and mental health consequences: systematic review of the current evidence. *Brain Behav. Immun.* 89 531–542. 10.1016/j.bbi.2020.05.048 32485289 PMC7260522

[B93] Wagenmakers E.-J. Marsman M. Jamil T. Ly A. Verhagen J. Love J. (2018). Bayesian inference for psychology. part I: theoretical advantages and practical ramifications. *Psychon. Bull. Rev.* 25 35–57. 10.3758/s13423-017-1343-3 28779455 PMC5862936

[B94] Wessel J. R. Aron A. R. (2017). On the globality of motor suppression: unexpected events and their influence on behavior and cognition. *Neuron* 93 259–280. 10.1016/j.neuron.2016.12.013 28103476 PMC5260803

[B95] Wessel J. R. Conner C. R. Aron A. R. Tandon N. (2013). Chronometric electrical stimulation of right inferior frontal cortex increases motor braking. *J. Neurosci.* 33 19611–19619. 10.1523/JNEUROSCI.3468-13.2013 24336725 PMC3858630

[B96] Zhang R. Geng X. Lee T. M. C. (2017). Large-scale functional neural network correlates of response inhibition: an fMRI meta-analysis. *Brain Struct. Funct.* 222 3973–3990. 10.1007/s00429-017-1443-x 28551777 PMC5686258

[B97] Zhang Y. Ma Z. F. (2020). Impact of the COVID-19 pandemic on mental health and quality of life among local residents in liaoning province. China: A cross-sectional study. *Int. J. Environ. Res. Public Health* 17:2381. 10.3390/ijerph17072381 32244498 PMC7177660

[B98] Zigmond A. S. Snaith R. P. (1983). The hospital anxiety and depression scale. *Acta Psychiatr Scand* 67 361–370. 10.1111/j.1600-0447.1983.tb09716.x 6880820

